# Platelet Hyaluronan Synthase 3 Regulates Thrombin Signaling and Adhesion to Fibrinogen Under Venous Shear

**DOI:** 10.1002/pgr2.70035

**Published:** 2025-07-31

**Authors:** Kimberly A. Queisser, Lydia Smith, Gabriel Leung, Nansy Albtoush, Rebecca A. Mellema, Aaron C. Petrey

**Affiliations:** 1 University of Utah Molecular Medicine Program, Salt Lake City, Utah, USA; 2 Department of Pathology, Division of Microbiology & Immunology, University of Utah School of Medicine, Salt Lake City, Utah, USA; 3 Division of Gastroenterology, Hepatology and Nutrition, University of Utah, Salt Lake City, Utah, USA

**Keywords:** Hematopoiesis, Hyaluronan, Megakaryocyte, Platelet, Thrombin

## Abstract

Hyaluronan (HA) is an essential glycosaminoglycan with supportive roles in hematopoiesis. HA is synthesized by hyaluronan synthases (HAS1, HAS2, and HAS3) and plays a crucial role in cellular signaling and extracellular matrix interactions. Megakaryocytes (MKs), the platelet progenitor cell, express only HAS2 and HAS3, and dysregulated metabolism of HA by MKs leads to thrombocytopenia. To unravel the contribution of HAS3 in platelet function, Using HAS1/3 knockout (dKO) mice, we demonstrate that thrombin-mediated activation is significantly impaired, whereas collagen-dependent activation remains intact. Functional assays indicate that platelet aggregation, integrin α_IIb_β3 activation, and granule secretion are reduced in HAS1/3 KO platelets. However, tail bleeding times remain normal, suggesting that primary hemostasis is not severely affected. Under flow conditions, dKO platelet adhesion to fibrinogen is deficient under venous shear, while adhesion under arterial shear is unaffected. Mechanistically, these impairments correlate with reduced phosphorylation of AKT (p-AKT), while phosphorylation of PLCγ (p-PLCγ) remains preserved, suggesting that expression of HAS3 selectively regulates platelet function. These findings highlight HA synthesis as a novel regulator of thrombin-induced platelet activation and suggest that HAS enzymes may be previously unknown modulators of hemostatic and thrombotic responses.

## 1 | Introduction

Glycosaminoglycans (GAGs) are essential macromolecular components of all mammalian tissues where they exert important structural and regulatory functions. Hyaluronan (HA) is unique among GAGs as it is not attached to a core protein, is synthesized at the plasma membrane, and consists of an unsulfated sequence of repeating disaccharide units of glucuronic acid and *N*-acetyl-d-glucosamine joined by alternating β1,4 and β1,3 linkages which form a negatively charged hydrophilic polymer. Many of the biochemical and biophysical properties of HA are regulated by its size distribution and in healthy tissues, naturally occurring HA ranges between sizes of low molecular weight (LMW) < 200 kDa to high-molecular weight (HMW) HA of over 1000 kDa [1]. HA is produced by three transmembrane hyaluronan synthases (HAS1, HAS2, and HAS3), each with varying tissue- and cell-type expression patterns and biosynthetic rates. HA production by the HASs is dependent upon uridine diphosphate (UDP) glucuronic acid (UDP-GlcUA) and UDP N-acetylglucosamine (UDP-GlcNAc) as substrates and is accordingly regulated by metabolic state [2]. HA displays a variety of biological activities, and directs cell signaling and developmental programs by engagement with one or several cell surface receptors [3].

Despite the well-characterized functions of HA, its role in blood cell production and function is not well described on a mechanistic level. In the bone marrow (BM), HA is a key component of the hematopoietic niche, where it influences the behavior and function of various progenitor cells. Prior studies demonstrated that HA is a key component of the extracellular matrix (ECM) of the bone marrow, where it broadly regulates several cellular functions including adhesion, migration, and retention, and cytokine production through interactions with HA receptor CD44. Inhibition of HAS activity and HA production significantly affects the hematopoietic system, and BM hematopoietic stem and progenitor cells (HSPCs) isolated from HAS1/2/3 triple knockout (KO) mice fail to establish hematopoiesis in vitro [4]. Inhibition of HA synthesis by 4-methylumbilliferone (4MU) also profoundly inhibits in vitro models of hematopoiesis [5]. Addition of exogenous HA can partially rescue cell function, and hematopoietic activity of BM cells from these mice is rescued by coculture in the presence of HA-expressing stromal cells. Further, HA cooperates with stromal derived factor 1 (SDF-1) in trafficking of CD34+ progenitor cells to the BM (REFX 15070674), and contributes to spatial distribution of HSCs after transplantation in a HAS3-dependent manner (REFX 21673348).

Megakaryocytes (MKs) are the largest of the hematopoietic cells and are essential for producing platelets. MKs assemble and release platelets by extending lengthy proplatelet structures through the BM vasculature and into the bloodstream. MKs form a network of tubular invaginations of the plasma membrane to give rise to nascent platelets [6]. Unlike other progenitor cell types in the BM, MKs are characterized by expression of HAS2 and HAS3 and the intracellular localization of HA and HA-binding receptors [7, 8]. Our previous studies were the first to demonstrate a novel role for HA depolymerization by hyaluronidase-2 (HYAL-2) in megakaryocyte maturation and thrombopoiesis. Loss of Hyal-2 resulted in to pronounced macrothrombocytopenia, significantly increased HA within the marrow, aberrant MK maturation, and reduced pro-platelet formation, which are rescued by reconstitution of hyaluronidase activity in MKs [8].

Platelets are critical for hemostasis and wound healing with increasingly appreciated roles in inflammation [9], and their function is tightly regulated by the microenvironment in which they are produced [10]. Platelets promote thrombus formation through three key activities: activation, aggregation, and adhesion. These essential functions of platelets are engaged upon recognition of extracellular stimuli such as soluble agonists (e.g. thrombin, which activates the G-protein coupled proteinase-activated receptors (PAR)) and adhesive matrix proteins (e.g. collagen, which activates the glycoprotein VI (GPVI), an immunoreceptor tyrosine-based activation motif (ITAM) receptor) [11-13]. Synthesis of HA increases dramatically during inflammatory disease, and in many settings such as sepsis, inflammatory bowel disease, cancer, and COVID-19, circulating HA is reported to increase between 2- and 10-fold in circulation [14-19]. The consequences of altered HA content on platelet function are not well defined. Rare Hyal-2 mutations in humans are associated with increased platelet reactivity to agonists and thrombosis risk [20], suggesting HA levels within the bone marrow might impact platelet reactivity.

In this study, we present evidence that HAS1/3 double knock out (dKO) mice exhibit agonist-dependent differences in platelet function. Our data show that reduced HA content within the BM due to ablation of HAS1 and HAS3 does not significantly impact platelet number, size, or circulating half-life. However, platelets produced by HAS1/3 dKO mice show altered functional responses. Isolated, washed platelets from dKO mice show normal activation, aggregation, and adhesion under shear in response to collagen via GPVI, but are hypo-responsive to aggregation, and activation by either thrombin or PAR4-activating peptide (PAR4AP) and further exhibit reduced adhesion to fibrinogen under venous shear. These functional deficits in integrin α_IIb_β3 activation and adhesion are not due to changes in platelet surface receptor expression levels but by impaired Akt-signaling downstream of thrombin. Collectively, these data reveal a previously unknown role for HA synthases in platelet functional responses.

## 2 | Materials and Methods

### 2.1 | Mouse Strains

All animal experiments were conducted according to the animal welfare protocol approved by the Institutional Animal Care and Use Committee (IACUC) of the University of Utah. All mice were at adult age and c57Bl/6 background strain housed and bred in specific pathogen-free (SPF) microisolator cages on standard Teklad irradiated chow and acidified water in the AALAC certified Office of Comparative Medicine. Control, wild-type C57bl/6 J used for experiments or breeding with HAS1/3 double KO mice were purchased from Jackson Labs (Stock 000664). HAS1/3 dKO mice were generated as previously described (PMC4581575).

Genotypes were confirmed by PCR using genomic DNA prepared with Qiagen (Alameda, CA) and an Epicentre Technologies (Madison, WI) PCR Kit, performed on a Thermo Scientific HyBaid PCR Express machine (Wilmington, DE) under the following cycling conditions: 95°C for 30s, 65°C for 30s, and 72°C for 1 min, repeated for 35 cycles. The PCR products for each primer set were as follows: a 341 bp product for the HAS1 wild-type allele (mHAS1s: 5′GACGTTCTGGCCCTGGTCCTAC 3′ and mHAS1as: 5′GGGCTCTACTGCTGCTTGGAGG 3′) and a 320 bp product for the HAS3 wild-type allele (mHAS3s: 5′GGAAGCAGGCATAGGTAGCCTTG 3′ and mHAS3as: 5′TGATCGGCACCTTACCAACCGAG 3′). The antisense primer specific to the null allele (PGKpromAS-1: 5′GAGGCCACTTGTGTAGCGCCAAG 3′) was paired with the sense primers to confirm single (HAS1KO 331 bp, HAS3KO 320 bp) and double-null genotypes in mice.

### 2.2 | Mouse BM Histologic Analysis

Mouse femurs were fixed for 48 h in 10% neutral buffered formalin, and bones were then decalcified for 10 days in 12% EDTA (pH 8.0) at room temperature with constant rotation. EDTA was changed twice daily. Tissues were paraffin-embedded, sectioned, and stained with HA-binding protein (HABP) as previously described [8].

### 2.3 | Immunohistochemistry

Bone marrow–derived megakaryocytes (MKs) were enriched using a bovine serum albumin gradient, fixed with 4% paraformaldehyde, and centrifuged onto poly-l-lysine–coated coverslips at 250 × g for 10 min. The cells were then permeabilized with cold methanol for 5 min. After fixation, samples were washed with phosphate-buffered saline (PBS) and blocked with 2% fetal bovine serum in Hanks’ balanced salt solution for 1 h. Cells were incubated with biotinylated hyaluronan-binding protein (HABP; EMD Millipore, Billerica, MA) and an anti-von Willebrand factor (VWF) antibody (Abcam). Following washes, samples were incubated with an Alexa Fluor-conjugated secondary antibody for 45 min at room temperature and washed again. Vectashield Mounting Medium with DAPI was applied for nuclear staining. Images were acquired using a Leica TCS SP5 II confocal/multiphoton high-speed upright microscope (Leica Microsystems) equipped with an HCX PL APO 63X/1.4NA oil immersion objective, Leica HyD system detector, and Leica LAS AF software version 2.6. Image processing was performed using Fiji version 2.0 (http://fiji.sc) and ImageJ version 1.47 (NIH, Bethesda, MD; http://imagej.nih.gov/ij).

### 2.4 | Murine Platelet Isolation

Whole blood from 12-week-old C57bl/6 J mice was collected via cardiac puncture and mixed with 300 μL of 3.2% sodium citrate (pH 7.4). The blood was then diluted to a total volume of 2 mL with pre-warmed (37°C) PIPES-saline glucose buffer (PSG, 5 mM PIPES, 145 mM NaCl, 4 mM KCl, 50 μM Na2HPO4, 1 mM MgCl2·6H2O, 5.5 mM glucose, pH 6.8) and centrifuged at 200 × g for 7 min without braking to obtain platelet-rich plasma (PRP). The PRP was carefully transferred to fresh Eppendorf tubes, and PGE1 was added at a final concentration of 282.67 ng/mL. The PRP was then centrifuged at 400 × g for 10 min at room temperature. The resulting platelet pellet was resuspended in PSG containing PGE1 and 0.01 U/mL apyrase and washed twice. Finally, the platelets were resuspended in M199 or HEPES-Tyrode's buffer for subsequent experiments.

### 2.5 | Platelet Aggregation

Platelet aggregation was measured by light transmission aggregometry in washed murine platelets (2 × 10^8^ platelets/mL) under stirring conditions at 37°C using a PAP-8E Platelet Aggregometer (Bio/Data Corporation). Platelets were stimulated with indicated agonists and the change in light transmission measured in response to collagen (ChronoLog), thrombin (ChronoLog), or 2MeSADP (Sigma), at indicated concentrations. For ADP-dependent aggregation assays, fibrinogen was added to washed platelets at a concentration of 2 mg/mL.

### 2.6 | Tail-Bleeding Times

Tails of anesthetized mice (isoflurane) between 6 and 10 weeks of age were transected 4 mm from the tip with a scalpel and the remaining tail was immersed in 37°C saline. The time until bleeding stopped for more than 1 min was observed and recorded.

### 2.7 | Platelet Shear Flow Assays

Platelet adhesion was measured under shear conditions using Vena8 Fluoro+ (Cellix) microfluidic chips coated overnight with collagen (20ug/mL), or fibrinogen (100ug/mL). Flow cells were blocked for 1 h at 37°C with 0.1% BSA in PBS. Isolated platelets at 6 × 10^8^plts/mL were dyed with 0.67uM DIOC6 [3] for 15 min at 37 °F. 2.5 × 10^8^ dyed platelet were mixed with red blood cells making up 40% of the final volume. Platelets were then aspirated at −50 Dynes/cm^2^ for arterial and −4 Dynes/cm^2^ for venous shear, and imaged using an EVOS FL auto microscope at 20× magnification. Recordings of the flow chamber were analyzed in Fiji at multiple timepoints.

### 2.8 | Flow Cytometry

To measure bone marrow megakaryocytes, marrow from 1 femur and 1 tibia per mouse was extracted by cutting one end of the femur and centrifuging marrow into sterile 1.7 mL tubes at 5000 rpm for 30 s. Pellets were resuspended and erythrocytes were lysed from each sample using ACK lysis buffer, and samples were diluted in 5 mM EDTA in PBS, filtered through a 100 micron filter, and 50uL of CountBrite counting beads (52,000 beads) were added to each strainer. Cells were washed twice in 5 mM EDTA in PBS, pelleted at 400×g for 5 min, and resuspended in 600uL of 5 mM EDTA in PBS. 200 uL of BM cells were stained in a 96-well plate with the following antibodies: CD16/32 PGP (Biolegend clone: 93, Fc Block), F4/80-biotin (Biolegend clone: BM8), FcER1a-biotin (Biolegend clone: MAR-1), CD3-biotin (Biolegend clone: 145-2C11), Ter119-biotin (Biolegend clone: TER119), NK1.1-biotin (Biolegend clone: PK136), B220-biotin (Biolegend clone: RA3-6B2), CD11b-biotin (Biolegend clone: M1/70), Gr1-biotin (Biolegend clone: RB6-8C5), CD45 BUV395 (Biolegend clone: 30-F11), Kit BV605 (Biolegend clone: ACK2), CD41a PE-Cy7 (Biolegend clone: MWReg30), CD42 Alexa Fluor 488 (Emfret Cat. No. X488). 4’,6-Diamidino-2-Phenylindole, Dihydrochloride (DAPI, 300 nM, Invitrogen) was used to exclude dead cells.

To assess platelet activation, washed platelets were diluted in M199 to a concentration of 2 × 10^7^ platelets/mL. 100 μL of platelet solution was incubated with 2 μL of CD41a antibody, 5 μL of JonA antibody, and 2 μL of CD62P antibody. Platelets were treated with or without agonists (PAR4AP, sequence=AYPGKF, GLBiochem), Convulxin, SantaCruz Biotech) and incubated in the dark for 15 min at 37°C. Following incubation, 200 μL of 1× fix-lyse buffer was added, and the surface expression of CD41 (α_IIb_), active integrin α_IIb_β3 (JonA), and CD62P (P-selectin). Intracellular signaling assays were performed by incubating platelets in the presence or absence of thrombin or convulxin and anti-CD41 (Biolegend) for 3 min before fixation with BD Phosflow Lyse/Fix buffer (BD Biosciences) according to manufacturer's protocol. Levels of p-AKT and p-PLCγ2 were measured using FITC-conjugated antibodies (BD Biosciences) and comparing the change in mean fluorescence intensity compared to isotype controls. Platelets were analyzed using a CytoFlex flow cytometer (Becton-Dickinson).

### 2.9 | Platelet Glycoprotein Surface Profiling

Platelet surface glycoproteins (GPs) were investigated by flow cytometry using washed platelets diluted in M199 to 2 × 10^7^plts/mL and 100 μl of murine platelet suspensions were incubated with 2 μl of fluorescently conjugated antibodies: GPIbα, GPIbβ, GPIX, GPV, GPVI (EMFRET Analytics), and CD41, CD61, CD29 (Biolegend) for 15 min. After incubation, reactions were stopped by addition of 200ul of 1x fix-lyse buffer and MFI of each receptor was analyzed using a CytoFlex flow cytometer.

### 2.10 | Platelet Counts and Turnover

Platelet counts and mean platelet volume measurements were performed using Hemavet 950 (Drew Scientific). Platelet circulating half-life was measured by in vivo by retro-orbital injection of 3.5 μg/mouse of Dylight488-GPIbβ (Emfret Analytics). Blood was collected at into 3.2% sodium citrate 3 h after injection to establish a baseline and at the time points indicated. Blood was stained with CD41-APC followed by fixation and lysis of red blood cells in fix-lyse buffer. The ratio of Dylight labeled to unlabeled platelets was analyzed by flow cytometry.

### 2.11 | Statistical Analyses

All experiments were performed at least in triplicate, and data are represented as means ± SEM. Data were analyzed with the *t*-test using Prism software version 6.0 (GraphPad Software Inc., La Jolla, CA). Differences were considered significant at *p* < 0.05.

## 3 | Results

### 3.1 | Multiple HA Synthases Contribute to Thrombopoiesis

Previous studies have shown that hematopoietic progenitors express all 3 HAS genes, while megakaryocytes and platelets exclusively express HAS2 and HAS3 [7]. Depolymerization of HA by HYAL-2 is an essential step for formation of the MK demarcation membrane and thrombopoiesis [8], but the specific contribution of HASs to platelet function is not known. Given that MKs only express HAS2 and HAS3, we turned to HAS1/3 double knock out (dKO) mice (preventing compensation in HAS1) to examine the role of HAS3 in megakaryocyte and platelet function.

**FIGURE 1 |:**
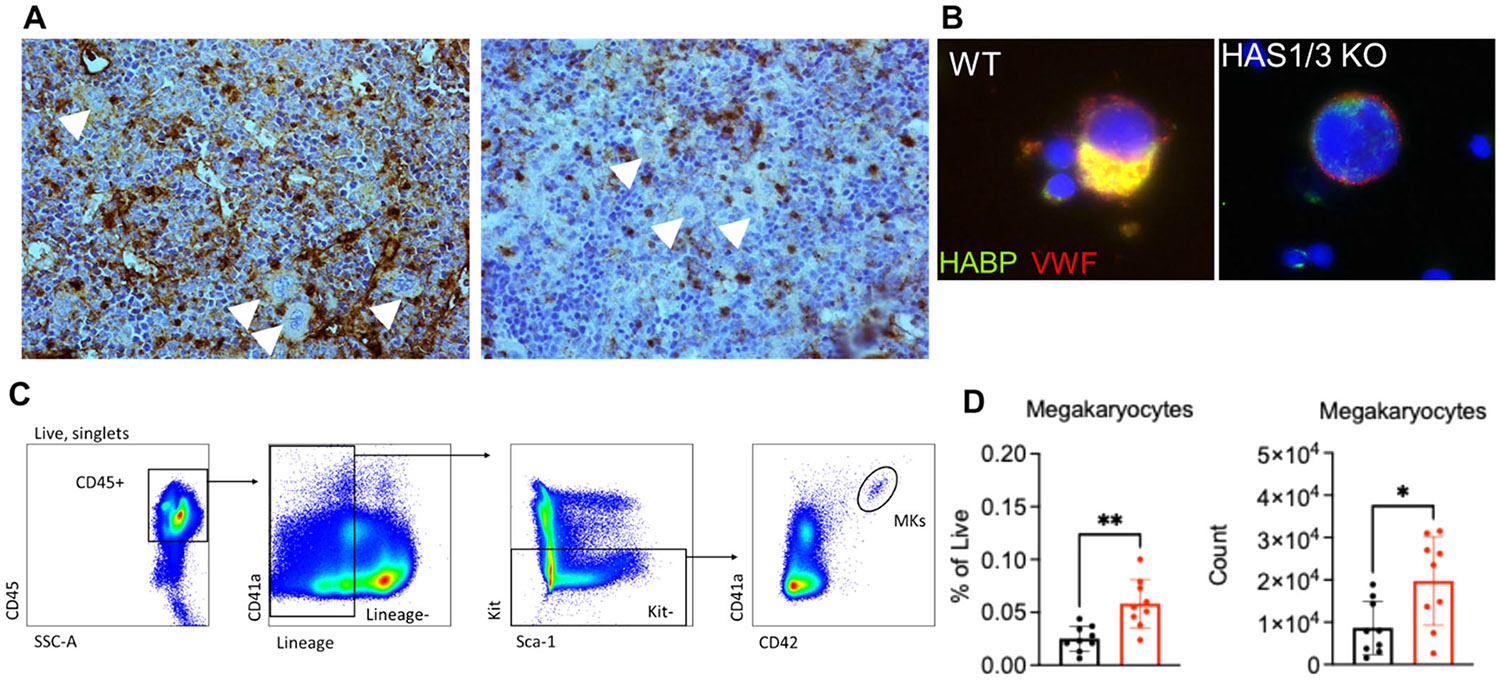
Reduced marrow HA has no gross defect in HAS1/3 dKO MKs and platelets. (A) Hyaluronan-binding protein (HABP) histochemical staining of wild-type (WT) and HAS 1/3 dKO femur BM sections. Arrows indicate megakaryocytes (MKs). (B) Representative immunofluorescence images of HA (green), vWF (red), and DAPI (blue). Scale bar = 25 μm. (C) Representative FACS plot showing gating strategy identifying MKs in live bone marrow cells. (D) Quantification of the frequency and cell counts of MKs in WT and dKO mice, *n* = 9 each, *p < 0.05 and **p < 0.01 using a paired Student's *t* test.

As a first step, we evaluated the quantity and localization of HA in the bone marrow of dKO mice by immunohistochemistry. While gross morphologic features appeared normal, the localization and quantity of HA distributed throughout the BM appeared significantly reduced in dKO mice compared with controls. The majority of HA appears widely distributed in the extracellular space, lining endothelial sinusoids, and appears punctate in some individual cells (including MKs) in control mice. In HAS dKO mice, extracellular and sinusoidal HA appears significantly reduced with only punctate, single cells staining strongly with HABP with MKs exhibiting reduced pericellular HA (Figure 1A). Localization of HA in mature MKs appears intracellular and colocalized with the maturation marker von Willebrand factor (vWF), which is present at MK cell surface and distributed through the DMS in control MKs (Figure 1B). By contrast, dKO MKs contain reduced levels intracellular HA, possibly owing to residual production by HAS2, and further displayed surface but not DMS localization of vWF. We next asked whether the reduction in marrow HA corresponded with changes in MK frequency or number by analyzing bone marrow cells by flow cytometry (Supporting Figure 1). Analysis of murine bone marrow MKs, defined as CD45+Lin-Kit-CD41a + CD42+ (Figure 1C) revealed that MKs are approximately twofold increased in the marrow of dKO mice as compared to control mice (Figure 1D).

**FIGURE 2 |:**
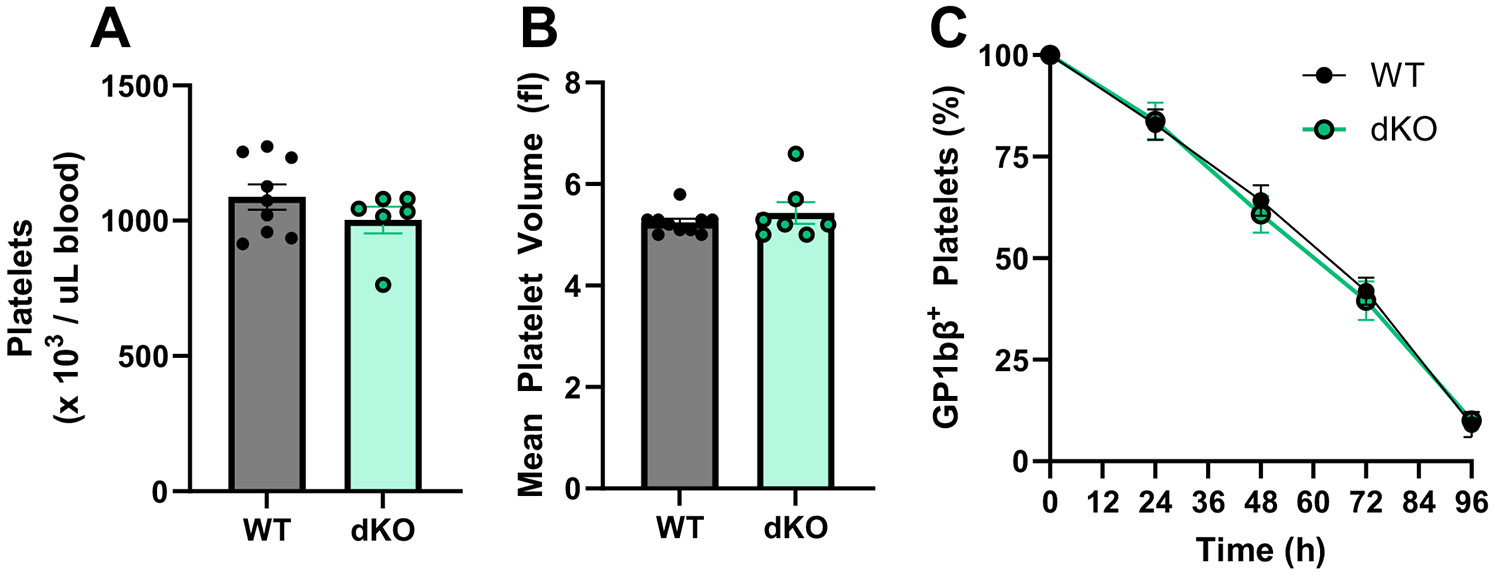
HAS 1/3 dKO mice have no overt defects in platelet counts, volume, or lifespan. Circulating platelet counts (A) and mean platelet volume (B) in whole blood. (C) Platelets were labeled in vivo and circulating platelet lifespan was measured by comparing the fraction of fluorescently labeled platelets of the total platelets by flow cytometry. Data are means ± SEMs for 4–6 mice per group.

As platelet count and volume may be influenced by MK maturity, we next examined whether reduced HA in the BM or MKs of dKO mice led to alterations in thrombopoiesis similar to HYAL2 KO mice [8]. Hematological analysis of platelet count (Figure 2A) and volume (Figure 2B) revealed no significant difference between genotypes. Further, using an an in vivo labeling strategy, we additionally found no significant difference in circulating platelet half-life (Figure 2C). Together, these data suggest that while global depletion of HA production in the BM inhibits hematopoiesis, HA synthases display some functional redundancy in the platelet lineage with respect to thrombopoiesis.

### 3.2 | Platelets from HAS dKO Mice Show Abnormal Hemostatic Responses

**FIGURE 3 |:**
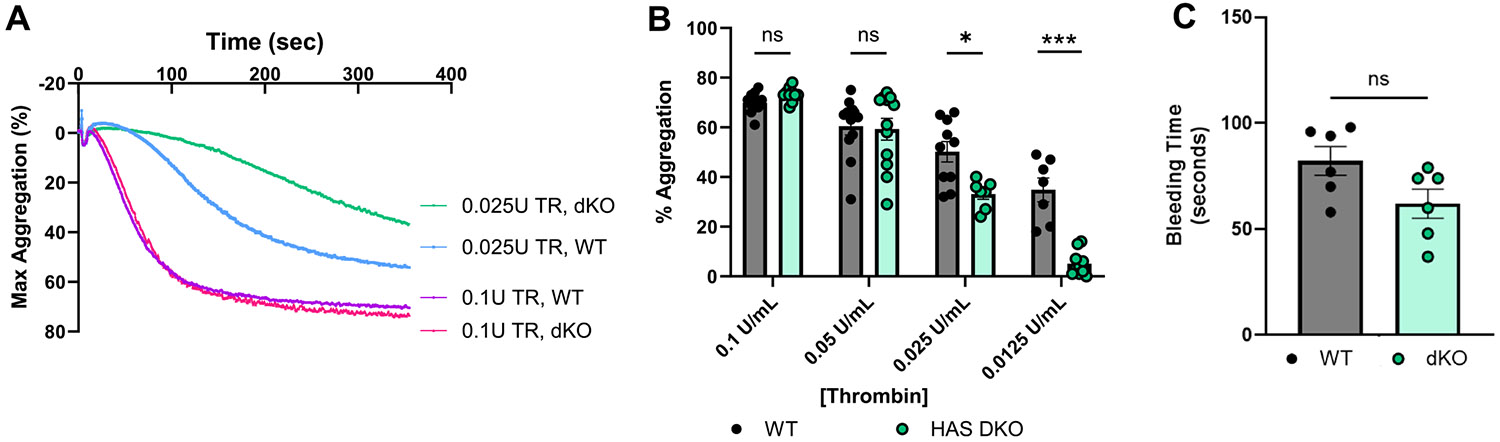
HAS1/3 dKO platelets have reduced aggregation responses to thrombin. The aggregation of washed platelets from control (WT) or HAS1/3 dKO mice was measured in the presence of increasing concentrations of thrombin, ranging from 0.1 U/mL to 0.0125 U/mL as measured by light-transmission aggregometry. (A) Representative aggregation tracings were compared for (B) maximal aggregation responses in control and dKO mice. (C) Tail bleeding times for WT and dKO mice. Data are means ± SEMs for 6–10 mice per group. *p < 0.05 and ***p < 0.001 using a paired Student's *t* test.

To assess whether disruption of HA synthases impact platelet function, we first evaluated washed platelets by light-transmission aggregometry. Treatment of platelets isolated from dKO mice showed comparable aggregation responses to control littermates (Figure 3A) at high doses of thrombin (0.1 U/mL or 0.05 U/mL). However, HAS1/3 deficiency resulted in significantly reduced platelet aggregation in response to submaximal (0.025 U/mL) or low dose (0.0125 U/mL) thrombin (Figure 3B). We next compared aggregation in response to collagen and found that platelets isolated from dKO mice exhibited normal collagen-mediated platelet aggregation (data not shown). To further test whether other GPCRs were affected, we measured platelet aggregation in response to maximal and low-dose ADP and observed no differences between genotypes (Supporting Figure 2A). Given that PAR signaling depends on Gq and G12/13 while P2Y12 depend on Gi, these data suggest loss of HAS3 may differentially affect GPCR-downstream signaling. To define the hemostatic phenotype of HAS1/3-deficiency in mice, we measured tail-transection bleeding time, a well-established model of hemostasis that depends primarily upon platelet binding to subendothelial collagen [21]. As shown in Figure 3C, dKO mice exhibited a trend toward reduced bleeding times as compared to control mice (*p* < 0.06). Taken together, our data show that HAS1/3 deficiency selectively impairs platelet function in response to thrombin and protease-activated receptor (PAR) activation, while collagen-dependent function through glycoprotein VI (GPVI) remains intact.

### 3.3 | Shear-Induced Adhesion to Fibrinogen but Not Collagen Is Defective in dKO Platelets

**FIGURE 4 |:**
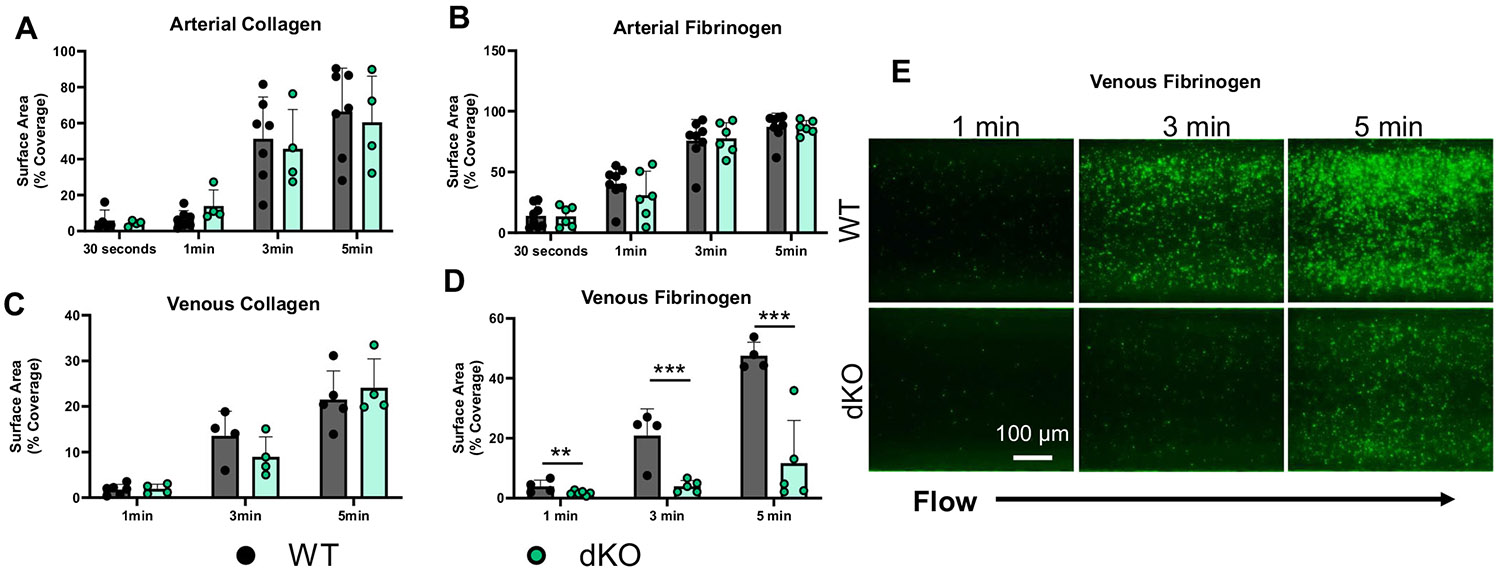
HAS1/3-deficient platelets show decreased thrombus formation on fibrinogen under venous shear conditions. Platelets from WT and dKO mice were aspirated over fibrinogen or collagen coated microfluidic channels. The percent of platelet surface area coverage was measured in arterial (A, B) (−50 Dynes/cm^2^) or (C, D) venous (−4 Dynes/cm^2^) shear conditions. (E) Representative image of thrombus formation on fibrinogen under venous shear. Data are means ± SEMs for 4–6 mice per group. Analysis performed using a paired Student's *t* test. *p < 0.05 ***p < 0.005 ****p < 0.001.

In response to vascular injury, blood flow marginates platelets in high concentrations near the vessel wall, where they initially interact with sub-endothelial collagen in the initial stages of thrombus formation by promoting platelet adhesion and activating platelets. Fibrinogen from circulation (or bound to HA on the vessel surface) binds to integrin α_IIb_β3 on the surface of activated platelets, crosslinks platelets together, enhancing platelet aggregation and strengthening the hemostatic plug. Given that dKO platelets showed reduced aggregation in response to thrombin, we next compared platelet-matrix interactions under arterial (−50 Dynes/cm^2^) or venous (−4 Dynes/cm^2^) shear conditions. Under conditions of arterial shear, we observed no significant differences in platelet adhesion to either collagen (Figure 4A) or fibrinogen (Figure 4B) between control and HAS1/3-deficient platelets. However, in settings of lower shear stress, while adhesion to collagen remained similar (Figure 3C), dKO platelets show significantly impaired adhesion to fibrinogen (Figures 4D, 4E). The findings indicate that HAS1/3 deficiency hinders platelet adhesion to fibrinogen under venous shear conditions, and this effect is surpassed by activation resulting from increased shear rates in arteries.

### 3.4 | HAS1/3-deficiency Impairs PAR-Dependent Integrin Activation and Granule Secretion

**FIGURE 5 |:**
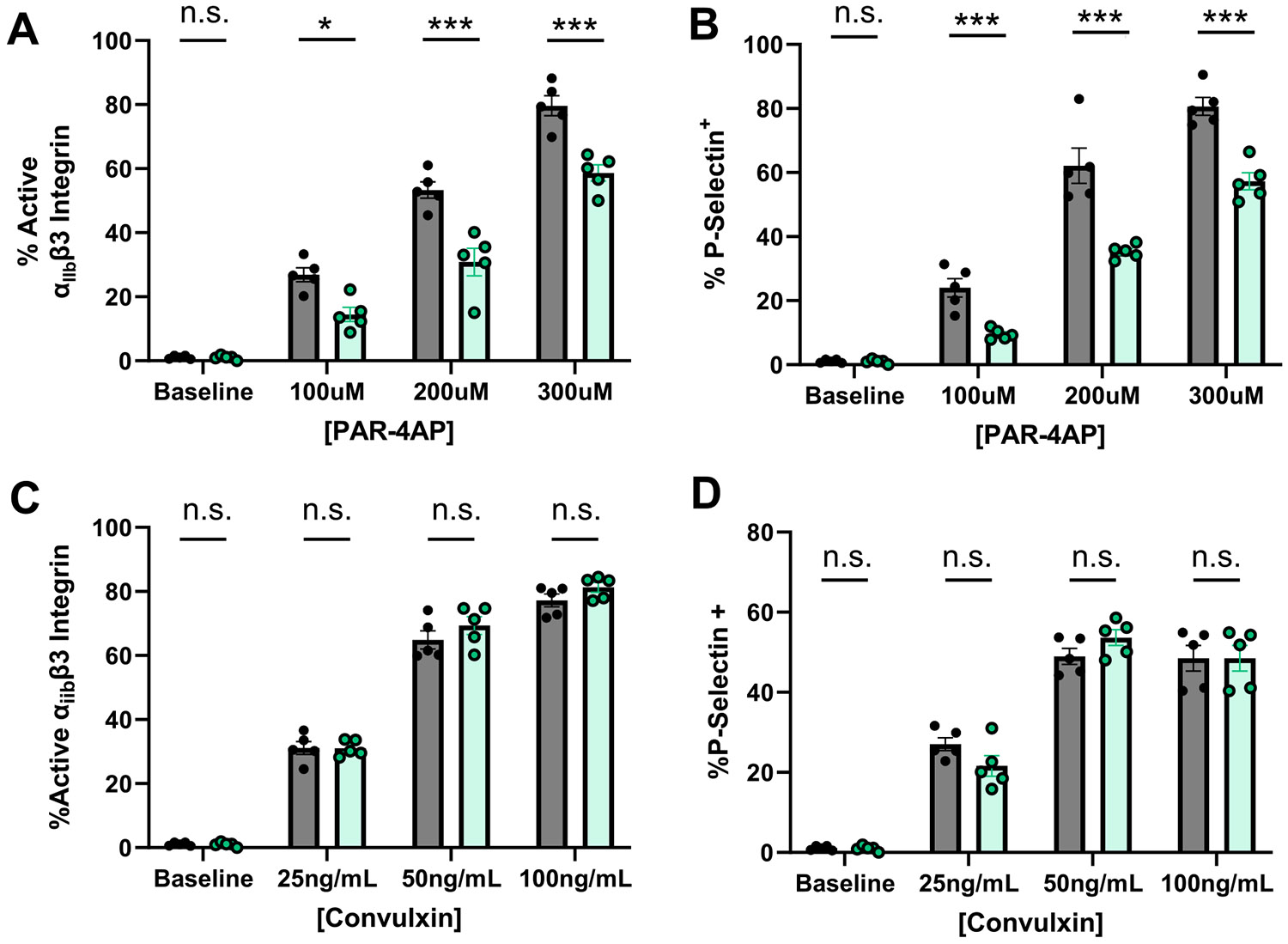
PAR-mediated activation responses are reduced in HAS1/3 KO platelets. Flow cytometry of agonist stimulated platelets isolated from WT or dKO mice. Washed platelets were stimulated with either PAR4-AP (A, B), or convulxin (C, D) at increasing concentrations and activation of integrin αIIBβ3 and granule secretion (surface P-selectin) were measured by flow cytometry. Data are means ± SEMs for 4–6 mice per group. Analysis performed using a paired Student's t test. *p < 0.05 **p < 0.01 ***p < 0.001.

Given that HAS dKO mice exhibited reduced aggregation in response to thrombin, but not collagen, and showed accelerated hemostatic function in a collagen-dependent bleeding model, we next evaluated agonist-induced platelet activation responses by flow cytometry (Supporting Figure 3). Treatment of dKO platelets with PAR4 agonist peptide (PAR4AP) revealed a significant reduction in the activation of integrin α_IIb_β3, which is responsible for binding fibrinogen and essential for platelet aggregation (Figure 5A). Similarly, secretion of platelet α-granules (as measured by surface P-selectin) was also reduced at all doses of PAR4AP (Figure 5B) in dKO mice. By contrast, activation of platelets with convulxin, a potent GPVI agonist, showed no difference between dKO mice and controls in both activation of integrin α_IIb_β3 (Figure 5C) and α-granule secretion (Figure 5D). Consistent with the observed agonist-dependent differences in hemostatic function we observed no significant differences in platelet activation in response to ADP (Supporting Figure 2B-C). Taken together, our results demonstrate that HAS1/3 deficiency selectively inhibits PAR-dependent platelet activation responses.

### 3.5 | Platelet Surface Receptor Expression Is Normal in dKO Platelets

Platelet surface glycoproteins and activating receptors play an essential function mediating critical steps in hemostasis, including adhesion, activation, and aggregation. Therefore, to determine whether altered expression of platelet surface receptors might explain hemostatic functional discrepancies observed in HAS1/3-deficient platelets, we next compared surface glycoprotein levels in washed platelets isolated from control or dKO mice by flow cytometry (Supporting Figure 4). Expression of major platelet surface glycoproteins, including the von Willebrand factor receptor complex subunits (GPIbα, GPIbβ, GPV, and GPIX), the collagen receptor GPVI, and both integrin β1 (CD29), β3 (CD61), α_IIb_ subunits (CD41), and PAR4 levels were not significantly different between genotypes.

### 3.6 | Decreased Phosphorylation of Akt in HAS1/3 dKO Platelets

**FIGURE 6 |:**
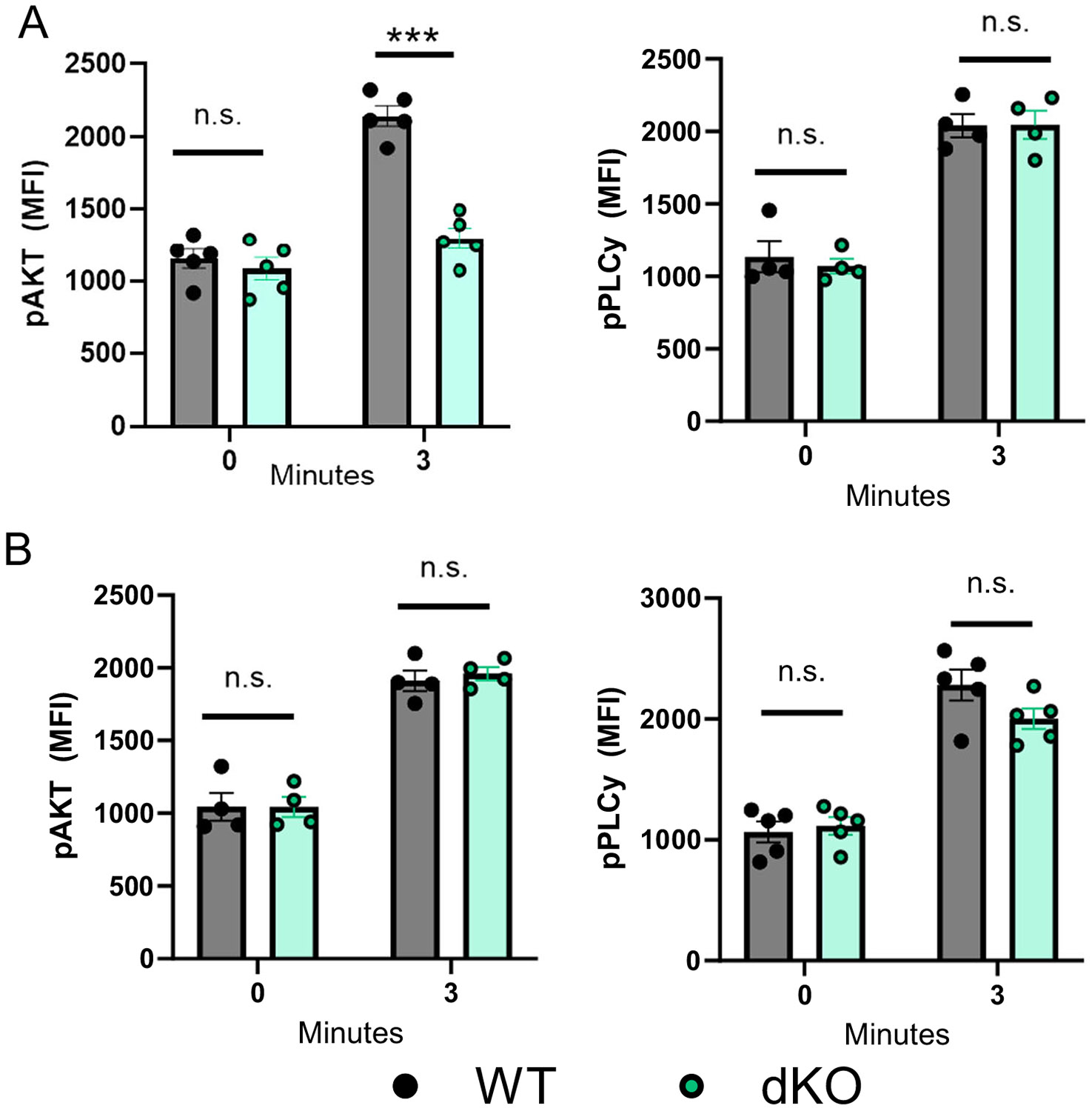
Agonist-induced Akt phosphorylation, but not PLCγ2, is reduced in HAS1/3 KO platelets). Platelets were isolated from control (WT) or dKO mice, washed, and stimulated with either (A) thrombin (0.025 U/mL) or (B) convulxin (25 ng/mL) to activate PAR or GPVI downstream signaling pathways. Basal (0 min) and activated (3 min) platelets were fixed, permeabilized, and intracellular levels of phospho-Akt and phospho-PLCγ2 levels were measured by flow cytometry. Data are means ± SEMs for 5 mice per group. Analysis performed using a paired Student's *t* test. ***p < 0.001.

Platelets integrins have a central functional role in distinct phases of platelet function during hemostasis and thrombosis, contributing to both platelet-platelet aggregation and platelet-matrix interactions. Critically, PAR-mediated platelet activation of α_IIb_β3 and adherence to fibrinogen depends upon the PI3K/Akt axis, whereas activation by GPVI is dependent upon PLCγ2 signaling [11]. Since dKO platelets showed reduced aggregation and activation responses to PAR-agonists and adhesion defects to fibrinogen under venous shear but normal responses to GPVI-agonists, we hypothesized that Akt signaling was affected in dKO platelets. To examine the mechanism underlying platelet hypo-responsiveness to thrombin in dKO mice, we assessed the phosphorylation state of Akt and PLCγ2 by intracellular flowcytometry. We therefore compared p-AKT and p-PLCγ2 levels in washed platelets isolated from dKO and control mice at baseline (0 min) or after 3 min of stimulation of with submaximal doses of thrombin (0.025 U/mL) or convulxin (25 ng/mL). These analyses show that despite no difference in p-Akt or p-PLCγ2 levels at baseline, upon thrombin stimulation of PARs dKO platelets have significantly reduced activation of p-Akt while exhibiting normal activation of p-PLCγ2 as compared to controls (Figure 6A). By contrast, in response to stimulation with convulxin, dKO platelets exhibit normal activation of p-Akt and p-PLCγ2 activation downstream of GPVI. Together, these findings suggest that HAS1/3-deficiency selectively impairs PAR-signaling in platelets.

## 4 | Discussion

The findings reported in this study demonstrate a novel role for hyaluronan synthases as regulators of platelet function in an agonist-specific manner. Our data show that genetic ablation of HAS1/3 modulates platelet responses differently depending on the stimulus, highlighting a previously unrecognized regulatory mechanism in platelet and megakaryocyte biology. First, the platelet functional data demonstrates that deletion of HAS1/3 results in a phenotype that is subtle, where global reduction of HA within the bone marrow does not appear to impact platelet count, volume, or circulating half-life. However, upon examination of platelet functional responses, HAS1/3 dKO mice show reduced hemostatic response to submaximal doses of thrombin (a GPCR-dependent agonist) while retaining normal function in response to collagen (an ITAM-dependent agonist) sufficient to retain primary hemostasis. Second, our data show that the underlying mechanism of this impaired response to thrombin is due to attenuated signaling downstream of PAR-activation resulting in reduced phosphorylation of Akt. Importantly, HAS1/3 dKO platelets retain normal responses to GPVI agonists downstream phosphorylation of PLCγ2 was not significantly different from controls, indicating agonist-specific signaling differences in dKO platelets. Earlier data from a number of laboratories, including our own, have shown that HYAL-2 regulates platelet function in both humans and mice [8, 9, 14, 20, 22, 23], and the findings presented here are the first to show that loss of HAS3 regulates platelet functional responses.

Platelets can be activated through different receptor-mediated pathways, including the PAR pathway, driven by thrombin, and the GPVI pathway, activated by collagen. While both pathways lead to platelet activation, aggregation, and thrombus formation, they have distinct molecular mechanisms. Both pathways lead to platelet activation, but thrombin activation is more potent and involved in thrombus propagation, while GPVI activation is crucial for platelet adhesion to the damaged vessel wall. The interplay between these pathways ensures a tightly regulated hemostatic response while also contributing to pathological thrombosis if dysregulated. Our data demonstrates that the PAR-pathway is hyporesponsive upon knock-out of HAS1/3 as demonstrated by reduced integrin activation and granule secretion in washed platelets (Figure 5A-B). The initial steps of platelet signaling differ between PAR activation, which depends on GPCR signaling (through Gq, G12/13, Gi, PLCβ, and Akt) and GPVI which depends on ITAM-dependent tyrosine kinases (Src kinases, Syk, LAT, SLP-76, and PLCγ2). However, both pathways share key intracellular secondary messengers including inositol triphosphate (IP3), diacylglycerol (DAG), and Ca^2+^ release. Therefore, while p-AKT activation downstream of PARs is impaired in HAS1/3 dKO platelets (Figure 6), our data suggests that secondary messenger signaling is preserved, given normal activation and aggregation responses to collagen and convulxin (Figure 5C-D), and bleeding times (Figure 3C).

We show here that dKO platelets exhibit normal thrombus generation under conditions of arterial shear on either collagen or fibrinogen matrices, whereas under venous shear thrombus formation on fibrinogen is significantly reduced. In these settings, platelet receptor binding to ECM ligands triggers “outside-in” signals, such as when integrin α_IIb_β3 binds to fibrinogen. We observed that dKO platelets show normal adhesion under arterial conditions, where shear-dependent activation stimuli are strongest and multiple signaling pathways converge [24]. However, under lower shear forces in venous conditions, dKO platelets show a selective impairment of adhesion to fibrinogen but not to collagen. Surface receptor profiling experiments (Supporting Figure 4) indicated that both the αIIb (CD41) and β3 (CD61) subunits were present at normal levels in dKO platelets, ruling out differences in protein expression as an explanation for this phenotype. Currently, the initial signaling event found to occur following α_IIb_β3 “outside-in” activation by fibrinogen is the binding of the G protein subunit Gα13 to the cytoplasmic domain of the β3 integrin [25]. Therefore, it is plausible that the deficiency in inside-out signaling via thrombin and outside-in signaling upon adhesion to fibrinogen may be due to effects on Gα13 upstream of Akt in HAS1/3 dKO platelets.

The finding that aggregation in response to thrombin in HAS1/3 dKO mice was initially surprising given there were no obvious differences in circulating platelet parameters (Figure 2). By comparison, loss of HYAL-2 results in significant macrothrombocytopenia and dramatic accumulation of HA in the BM and MKs which impacts pro-platelet formation and increased platelet activation in response to the GPCR-agonist ADP [8, 9]. These data suggest that while HA within the BM is dramatically reduced in dKO mice, platelet counts are maintained possibly due to increased MK number (Figure 1D), unlike in HYAL-2 deficiency where failure to depolymerize HA impacts proplatelet production by MKs. Given that MKs express only HAS2 and HAS3, our data implicate HAS3 as a regulator of platelet functional responses, possibly at the level of transcription or maturation and thrombopoiesis MKs.

These studies raise interesting questions regarding the spatial regulation of HA and HA-interacting receptors in thrombopoiesis. Differentiation of HSPCs into MKs is characterized by differential regulation of HA receptors and while CD44 progressively decreases, the expression of RHAMM remains stable during megakaryopoiesis. However, ex-vivo expansion of MK precursors cultured in the presence or absence of soluble HA in the media has been demonstrated to have no discernable impact on maturation, signaling, or pro-platelet production by MKs [7]. By contrast, HYAL-2 deficient MKs which possess significantly elevated levels of intracellular HA fail to produce platelets in culture. In HAS1/3 dKO mice, HAS2 remains functional in MKs, and despite the dramatic reduction in extracellular HA in the BM platelet counts are normal. These observations suggest that extracellular HA within the BM microenvironment does not directly impact platelet production, but rather intracellular pools of HA can impact MK maturation. Therefore, these lines of data suggest that HA within the BM matrix contributes to several signaling cascades relevant to HSPCs (PI3K/Akt, Raf1, MEK, ERK1/2 etc.) through CD44 (and additional receptors) are likely to have downstream transcriptional effects on MKs. The specific receptors and downstream mechanisms by which intracellular HA contributes to megakaryopoiesis remain to be defined.

The implications of these findings extend beyond basic platelet biology to potential clinical relevance. The ability of HAS genes to modulate platelet activation in an agonist-dependent manner raises the possibility that targeting HAS enzymes could be a novel therapeutic strategy for preventing thrombosis while preserving hemostasis. Current antiplatelet therapies, such as P2Y12 inhibitors and aspirin, broadly suppress platelet activation and increase bleeding risk. In contrast, HAS-targeting strategies might provide a more selective approach, reducing platelet hyperactivity in pathological conditions while maintaining normal hemostatic function.

Despite these promising findings, our study has some limitations. First, while we demonstrate an association between HAS1/3 deficiency and platelet function, further mechanistic studies will be needed to elucidate the precise molecular interactions between HA and platelet receptors. Additionally, in vivo studies will be necessary to confirm whether pharmacological inhibition of HAS can effectively modulate thrombosis without compromising normal hemostasis given the consequences of HA depletion on HSPCs. Furthermore, the potential contribution of HA regulating developmental transcriptional pathways upstream of platelet production by MKs should be considered in future investigations.

In conclusion, our study establishes a novel link between HAS3 and platelet function, demonstrating that HA synthases exert a functional effect on platelet activation in an agonist-specific manner. These findings provide new perspectives on the regulation of platelet activity and open avenues for further research into HAS-targeted therapies for thrombotic disorders.

## Supplementary Material

Supplemental Figures

Additional supporting information can be found online in the Supporting Information section.

**Supplemental Figure 1:** Gating strategy for bone marrow flow cytometry. **Supplemental Figure 2:** HAS1/3 dKO platelets exhibit normal aggregation and activation responses to ADP-induced activation. **Supplemental Figure 3:** Gating strategy for platelet flow cytometry. **Supplemental Figure 4:** Comparison of platelet Surface Glycoprotein and Receptor levels.

## Data Availability

The data that support the findings of this study are available from the corresponding author upon reasonable request.

## References

[1] Petrey AC and de la Motte CA, “Hyaluronan, a Crucial Regulator of Inflammation,” Frontiers in Immunology 5 (2014): 101, 10.3389/fimmu.2014.00101. 24653726 PMC3949149

[2] Caon I, Parnigoni A, Viola M, Karousou E, Passi A, and Vigetti D, “Cell Energy Metabolism and Hyaluronan Synthesis,” Journal of Histochemistry & Cytochemistry 69, no. 1 (2021): 35–47, 10.1369/0022155420929772. 32623953 PMC7780193

[3] Day AJ, “Hyaluronan-Protein Interactions: Lilliput Revisited,” Proteoglycan Research 2, no. 4 (2024): e70007, 10.1002/pgr2.70007.

[4] Schraufstatter I, Serobyan N, DiScipio R, Feofanova N, Orlovskaya I, and Khaldoyanidi SK, “Hyaluronan Stimulates Mobilization of Mature Hematopoietic Cells but Not Hematopoietic Progenitors,” Journal of Stem Cells 4, no. 4 (2009): 191–202. 20720593 PMC2891032

[5] Goncharova V, Serobyan N, Iizuka S, , “Hyaluronan Expressed by the Hematopoietic Microenvironment Is Required for Bone Marrow Hematopoiesis,” Journal of Biological Chemistry 287, no. 30 (2012): 25419–25433, 10.1074/jbc.M112.376699. 22654110 PMC3408194

[6] Machlus KR and Italiano JE Jr., “The Incredible Journey: From Megakaryocyte Development to Platelet Formation,” Journal of Cell Biology 201, no. 6 (2013): 785–796, 10.1083/jcb.201304054. 23751492 PMC3678154

[7] Currao M, Malara A, Di Buduo CA, Abbonante V, Tozzi L, and Balduini A, “Hyaluronan Based Hydrogels Provide an Improved Model to Study Megakaryocyte-Matrix Interactions,” Experimental Cell Research 346, no. 1 (2016): 1–8, 10.1016/j.yexcr.2015.05.014. 26027944 PMC5071306

[8] Petrey AC, Obery DR, Kessler SP, Flamion B, and de la Motte CA, “Hyaluronan Depolymerization by Megakaryocyte Hyaluronidase-2 Is Required for Thrombopoiesis,” American Journal of Pathology 186, no. 9 (2016): 2390–2403, 10.1016/j.ajpath.2016.05.004. 27398974 PMC5012466

[9] Petrey AC, Obery DR, Kessler SP, Zawerton A, Flamion B, and de la Motte CA, “Platelet hyaluronidase-2 Regulates the Early Stages of Inflammatory Disease in Colitis,” Blood 134, no. 9 (2019): 765–775, 10.1182/blood.2018893594. 31262781 PMC6716076

[10] Guo L and Rondina MT, “The Era of Thromboinflammation: Platelets Are Dynamic Sensors and Effector Cells During Infectious Diseases,” Frontiers in immunology 10 (2019): 2204, 10.3389/fimmu.2019.02204. 31572400 PMC6753373

[11] Guidetti GF, Canobbio I, and Torti M, “PI3K/Akt in Platelet Integrin Signaling and Implications in Thrombosis,” Advances in Biological Regulation 59 (2015): 36–52, 10.1016/j.jbior.2015.06.001. 26159296

[12] Stegner D and Nieswandt B, “Platelet Receptor Signaling in Thrombus Formation,” Journal of Molecular Medicine (Berl) 89, no. 2 (2011): 109–121, 10.1007/s00109-010-0691-5. 21058007

[13] Estevez B and Du X, “New Concepts and Mechanisms of Platelet Activation Signaling,” Physiology (Bethesda, Md.) 32, no. 2 (2017): 162–177, 10.1152/physiol.00020.2016. 28228483 PMC5337829

[14] Queisser KA, Mellema RA, Middleton EA, , “COVID-19 Generates Hyaluronan Fragments That Directly Induce Endothelial Barrier Dysfunction,” JCI Insight 6, no. 17 (2021), 10.1172/jci.insight.147472. PMC8492325 34314391

[15] Becker BF, Jacob M, Leipert S, Salmon AHJ, and Chappell D, “Degradation of the Endothelial Glycocalyx in Clinical Settings: Searching for the Sheddases,” British Journal of Clinical Pharmacology 80, no. 3 (2015): 389–402, 10.1111/bcp.12629. 25778676 PMC4574825

[16] Benatti MN, Fabro AT, and Miranda CH, “Endothelial Glycocalyx Shedding in the Acute Respiratory Distress Syndrome After Flu Syndrome,” Journal of Intensive Care 8 (2020): 72, 10.1186/s40560-020-00488-7. 32974033 PMC7503444

[17] Lennon FE and Singleton PA, “Hyaluronan Regulation of Vascular Integrity,” American Journal of Cardiovascular Disease 1, no. 3 (2011): 200–213. 22254199 PMC3253523

[18] Schmidt EP, Li G, Li L, , “The Circulating Glycosaminoglycan Signature of Respiratory Failure in Critically Ill Adults,” Journal of Biological Chemistry 289, no. 12 (2014): 8194–8202, 10.1074/jbc.M113.539452. 24509853 PMC3961648

[19] Smart L, Macdonald SPJ, Burrows S, Bosio E, Arendts G, and Fatovich DM, “Endothelial Glycocalyx Biomarkers Increase in Patients With Infection During Emergency Department Treatment,” Journal of Critical Care 42 (2017): 304–309, 10.1016/j.jcrc.2017.07.001. 28822340

[20] Eicher JD, Chen MH, Pitsillides AN, , “Whole Exome Sequencing in the Framingham Heart Study Identifies Rare Variation in HYAL2 That Influences Platelet Aggregation,” Thrombosis and Haemostasis 117, no. 6 (2017): 1083–1092, 10.1160/TH16-09-0677. 28300864 PMC7472427

[21] Bynagari-Settipalli YS, Cornelissen I, Palmer D, , “Redundancy and Interaction of Thrombin- and Collagen-Mediated Platelet Activation in Tail Bleeding and Carotid Thrombosis in Mice,” Arteriosclerosis, Thrombosis, and Vascular Biology 34, no. 12 (2014): 2563–2569, 10.1161/ATVBAHA.114.304244. 25278288 PMC4239193

[22] Albeiroti S, Ayasoufi K, Hill DR, Shen B, and de la Motte CA, “Platelet hyaluronidase-2: An Enzyme That Translocates to the Surface Upon Activation to Function in Extracellular Matrix Degradation,” Blood 125, no. 9 (2015): 1460–1469, 10.1182/blood-2014-07-590513. 25411425 PMC4342357

[23] de la Motte C, Nigro J, Vasanji A, , “Platelet-Derived Hyaluronidase 2 Cleaves Hyaluronan Into Fragments That Trigger Monocyte-Mediated Production of Proinflammatory Cytokines,” American Journal of Pathology 174, no. 6 (2009): 2254–2264, 10.2353/ajpath.2009.080831. 19443707 PMC2684190

[24] Li Z, Delaney MK, O'Brien KA, and Du X, “Signaling During Platelet Adhesion and Activation,” Arteriosclerosis, Thrombosis, and Vascular Biology 30, no. 12 (2010): 2341–2349, 10.1161/ATVBAHA.110.207522. 21071698 PMC3085271

[25] Gong H, Shen B, Flevaris P, , “G Protein Subunit Gα13 Binds to Integrin αIIbβ3 and Mediates Integrin “Outside-In” Signaling,” Science 327, no. 5963 (2010): 340–343, 10.1126/science.1174779. 20075254 PMC2842917

