# Supplementary material for: Platelet Hyaluronan Synthase 3 Regulates Thrombin Signaling and Adhesion to Fibrinogen Under Venous Shear
Source: Proteoglycan Res. Author manuscript; Available in PMC 2026 Jan 23. (PMC12826595; doi:10.1002/pgr2.70035)
Supplement: Supplemental Figures [file NIHMS2123807-supplement-Supplemental_Figures.pdf]

Supplemental Figures

Supplemental Figure 1

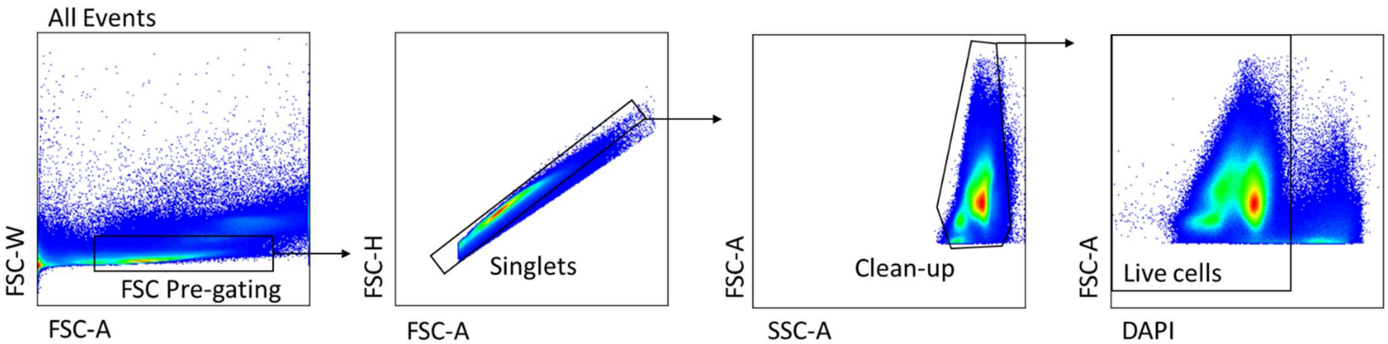

Supplemental Figure 1. **Gating strategy for bone marrow flow cytometry.**

Live bone marrow cells from 1 femur and 1 tibia were prepared for flow cytometry and identified on the basis of DAPI staining prior to subsequent gating to identify mature MKs.

Supplemental Figure 2

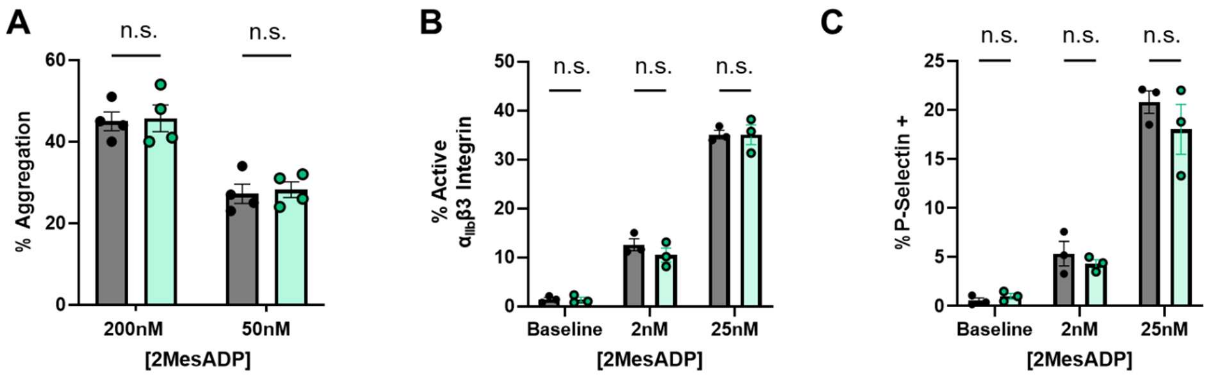

Supplemental Figure 2. **HAS1/3 dKO platelets exhibit normal aggregation and activation responses to ADP-induced activation.** The aggregation of washed platelets from control (WT) or HAS1/3 dKO mice was measured in the presence of increasing concentrations of 2MesADP, ranging from 200nM to 50nM in the presence of 1mg/mL fibrinogen as measured by light-transmission aggregometry. (A) maximal aggregation responses in control and dKO mice. Washed platelets from WT or dKO mice were stimulated with 2nM or 25nM 2MesADP and activation of integrin αIIBβ3 (B) and granule secretion (surface P-selectin) (C) were measured by flow cytometry. Data are means ± SEMs for 3-4 mice per group.

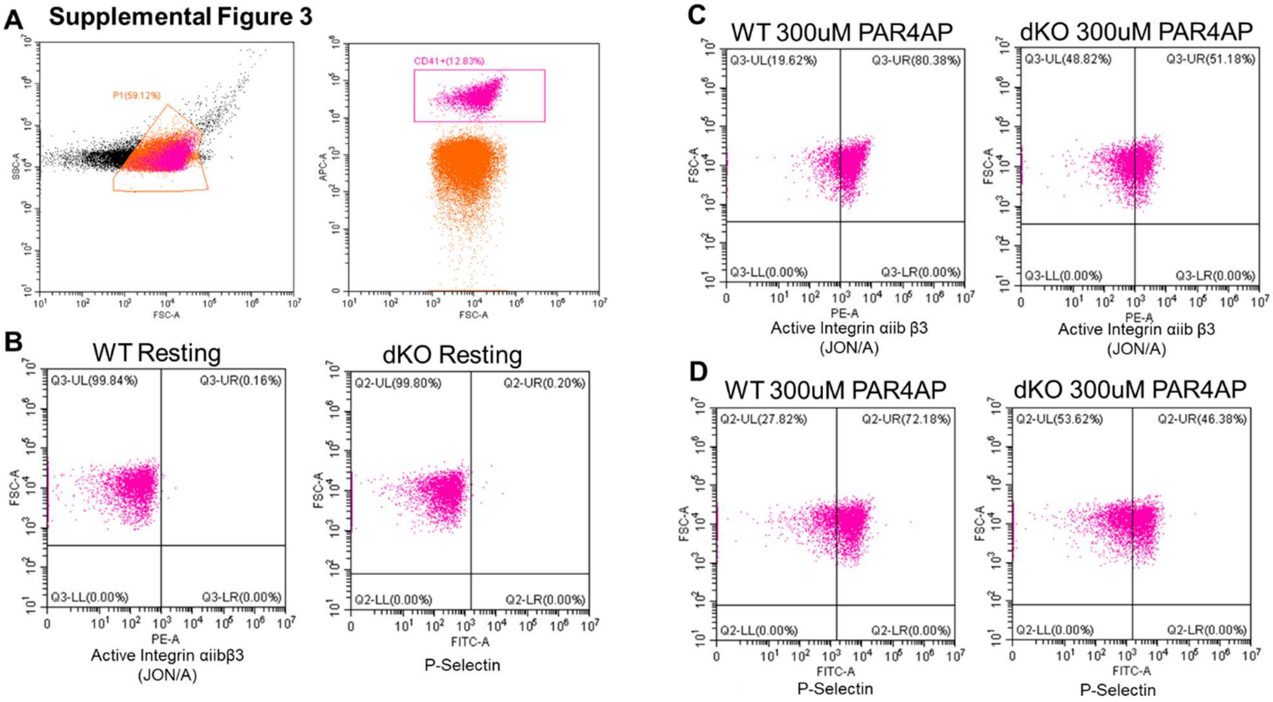

Supplemental Figure 3. **Gating strategy for platelet flow cytometry.**

(A) Platelets were identified on the basis of CD41 staining. (B) Activation of platelets was assessed by comparing the percent of (C) JonA and (D) P-Selectin positive platelets under baseline or stimulated (PAR4AP or Confluxin) conditions.

Supplemental Figure 4

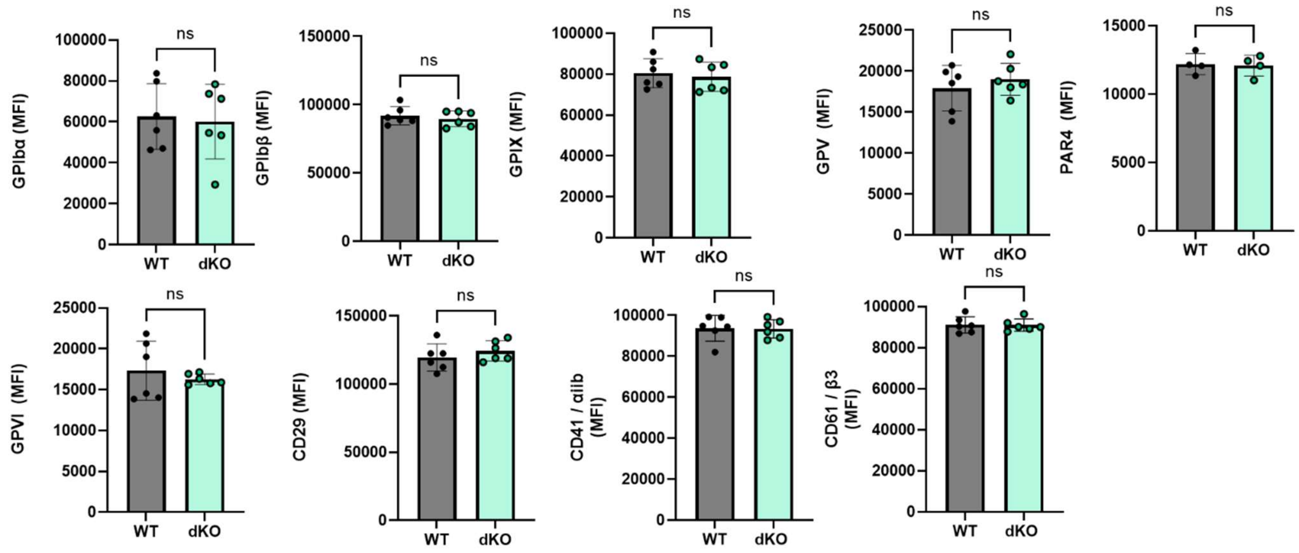

Supplemental Figure 4. **Comparison of platelet Surface Glycoprotein and Receptor levels.** Washed platelets were isolated from WT control and dKO mice and analyzed by flow cytometry for surface expression levels of key platelet glycoprotein and receptor receptors. MFI = mean fluorescence intensity. Data are means ± SEMs for 4-6 mice per group. Analysis performed using a paired Student’s t test.
